# The Developmental Trajectory of Brain-Scalp Distance from Birth through Childhood: Implications for Functional Neuroimaging

**DOI:** 10.1371/journal.pone.0024981

**Published:** 2011-09-21

**Authors:** Michael S. Beauchamp, Michelle R. Beurlot, Eswen Fava, Audrey R. Nath, Nehal A. Parikh, Ziad S. Saad, Heather Bortfeld, John S. Oghalai

**Affiliations:** 1 Department of Neurobiology and Anatomy, University of Texas Health Science Center, Houston, Texas, United States of America; 2 Department of Psychology, Texas A&M University, College Station, Texas, United States of America; 3 Department of Pediatrics, The Ohio State University, Columbus, Ohio, United States of America; 4 Center for Perinatal Research, The Research Institute at Nationwide Children's Hospital, Columbus, Ohio, United States of America; 5 Scientific and Statistical Computing Core, National Institute of Mental Health Intramural Research Program, Bethesda, Maryland, United States of America; 6 Department of Psychology, University of Connecticut, Storrs, Connecticut, United States of America; 7 Department of Otolaryngology Head and Neck Surgery, Stanford University, Palo Alto, California, United States of America; McGill University, Canada

## Abstract

Measurements of human brain function in children are of increasing interest in cognitive neuroscience. Many techniques for brain mapping used in children, including functional near-infrared spectroscopy (fNIRS), electroencephalography (EEG), magnetoencephalography (MEG) and transcranial magnetic stimulation (TMS), use probes placed on or near the scalp. The distance between the scalp and the brain is a key variable for these techniques because optical, electrical and magnetic signals are attenuated by distance. However, little is known about how scalp-brain distance differs between different cortical regions in children or how it changes with development. We investigated scalp-brain distance in 71 children, from newborn to age 12 years, using structural T1-weighted MRI scans of the whole head. Three-dimensional reconstructions were created from the scalp surface to allow for accurate calculation of brain-scalp distance. Nine brain landmarks in different cortical regions were manually selected in each subject based on the published fNIRS literature. Significant effects were found for age, cortical region and hemisphere. Brain-scalp distances were lowest in young children, and increased with age to up to double the newborn distance. There were also dramatic differences between brain regions, with up to 50% differences between landmarks. In frontal and temporal regions, scalp-brain distances were significantly greater in the right hemisphere than in the left hemisphere. The largest contributors to developmental changes in brain-scalp distance were increases in the corticospinal fluid (CSF) and inner table of the cranium. These results have important implications for functional imaging studies of children: age and brain-region related differences in fNIRS signals could be due to the confounding factor of brain-scalp distance and not true differences in brain activity.

## Introduction

Human brain and behavior both undergo remarkable changes during development, and there is intense interest in using functional neuroimaging techniques to better understand neurodevelopment. Many of these techniques, including fNIRS, EEG, MEG and TMS measure (or evoke) brain function using probes placed on or near the scalp. The physical distance between the scalp and brain is therefore a critical parameter for these techniques, especially for fNIRS. In fNIRS, and in related techniques such as event-related optical signaling [1], low-power near-infrared light is directed through the scalp and intervening tissues into the surface of the brain [2], [3]. Due to the differential absorption of specific wavelengths of near-infrared light by oxygenated and deoxygenated hemoglobin, concentration changes can be determined by measuring changes in the amount of near-infrared light sensed by detectors located on the scalp some distance from the near-infrared transmitter. Hence, fNIRS measures the same hemodynamic signal as measured with blood-oxygen level dependent functional magnetic resonance imaging (BOLD fMRI), the most popular method for examining human brain function [4], [5]. However, unlike fMRI, fNIRS depends on the transmission of light through the scalp, skull, meninges and CSF. The spatial sensitivity profile of fNIRS can be characterized as having a “banana” shape, with one end of the banana at the emitter, one end at the detector, and the body of the banana dipping down to sample the cortex [6]. The optimal placement of the detector and emitter therefore depends on the depth that light must penetrate: if the emitter and detector are close, more light will travel from the emitter to the detector, but none will travel through the brain; if the emitter and detector are distant, little light will reach the detector, resulting in poor signal-to-noise ratio. Our study was spurred by our experience in recording responses from auditory cortex in children with fNIRS [7]. In order to determine the optimal emitter-detector distance, we wanted to establish the distance between the brain and scalp for the different aged children in our study population. While there are published studies of brain-scalp distance in adults [8], [9], [10] and children [11], [12], we could find little information on how brain-scalp distance changes during development. To fill this gap, we examined MRI scans from 71 healthy children ranging in age from 1 day to 12 years old. We hypothesized that there would be significant differences between ages, with younger children having reduced brain-scalp distances; and significant differences between brain areas, with greater brain-scalp distances in some regions relative to others.

## Methods

Experiments were conducted in accordance with the Institutional Review Board of the University of Texas Health Science Center at Houston. Written informed consent was obtained from the guardian of each subject, and assent from the child subject if appropriate, prior to data collection. Information about scalp-brain distance was extracted from T1-weighted anatomical MRI images collected from each subject. The total subject population (*n* = 71) was assembled from 3 datasets.

### Dataset 1

The first dataset consisted of fourteen healthy full-term newborns (mean age 1.9 d; mean birth weight 3183 g, mean birth length 50.1 cm). The newborns were scanned using the 3 tesla whole-body MRI scanner (Phillips Medical Systems, Bothell, WA) at the University of Texas Medical School at Houston using an 8-channel head coil. All children were scanned after feeding during natural sleep, without sedation. Silicone earplugs were used to reduce ambient scanner noise. Images were collected using a magnetization-prepared 180 degree radio-frequency pulses and rapid gradient-echo (MP-RAGE) sequence optimized for gray-white matter contrast. One hundred sagittal slices were collected (slice thickness 0.94 mm) with in-plane resolution of 0.703 mm (256×256 matrix).

### Dataset 2

The second dataset consisted of seventeen healthy children aged 6 to 12 years that were scanned using the 3 tesla scanner at the University of Texas Medical School at Houston using an 8-channel phased array head coil. Two MP-RAGE acquisitions were averaged to improve signal-to-noise ratio. One hundred and sixty sagittal slices were collected (slice thickness 1.0 mm) with in-plane resolution of 0.938 mm (256×256 matrix). External ear defender-type headphones were used to reduce ambient scanner noise.

### Dataset 3

The third dataset consisted of forty subjects aged 7 months to 6 years obtained from release 4 of the Pediatric MRI Data Repository created by the NIH MRI Study of Normal Brain Development [13]. This is a multi-site, longitudinal study of typically developing children, from ages newborn through young adulthood, conducted by the Brain Development Cooperative Group. A listing of the participating sites and a complete listing of the study investigators can be found at http://www.bic.mni.mcgill.ca/nihpd/info/participating_centers.html. T1-weighted images were acquired from each subject with slightly varying parameters, depending on the acquisition site. Axial images were acquired (slice thickness of 3.0 mm) with in-plane voxel sizes between 0.938 and 1.0 mm. Additional information about the protocols are available from http://www.NIH-PediatricMRI.org.

### Software Used for Data Analysis

All image analysis was conducted using *AFNI*, *SUMA* and other components of a freely available, open-source suite of programs widely used for performing MRI and fMRI analysis (http://afni.nimh.nih.gov) [14], [15], [16]. *3dSkullStrip* (a program in the AFNI and SUMA suite) extracts the brain from MRI images using a variation on a surface growing approach [17] along with edge detection and size heuristics. *3dSkullStrip* first corrects for gross spatial image non-uniformities and then expands a spherical surface iteratively until it envelops the brain [18]. Once the brain surface is found, it is progressively pushed outward and smoothed until it encounters the largest external gradient representing the scalp/air interface. *3dSkullStrip* was found to be robust in a recent comparison of brain extraction methods [18]. *IsoSurface* is a program that performs isosurface extraction from a volume dataset. In this case, the volume is a binary mask of the brain or skull, and the extracted surface represents the outer boundary of that mask using the marching cubes algorithm [19]. *SurfToSurf* projects data from nodes on one surface to nodes on another. Each node from surface 1 is mapped to a node on the mesh of surface 2 that is closest to the intersection of the surface normal at node 1 with the mesh of surface 2.

### Creation of Scalp Surface Models

**Figure 1:**
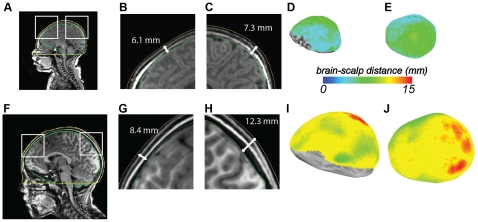
Whole-brain calculation of brain-scalp distance. A. Mid-sagittal slice through a full-term newborn brain (FT2009, age 2 days). The green line shows the reconstruction of the brain hull (bounding box of the cerebral cortex). The yellow line shows the reconstruction of the scalp surface. Both surfaces are truncated on the ventral surface of the brain (distance estimates were not computed for inferior brain regions where brain-scalp distance is ill-defined.) The white boxes show the areas enlarged in (B) and (C). B. Enlargement of anterior portion of mid-sagittal slice of newborn brain. The white line shows the distance between the reconstruction of the brain hull (green line) and the scalp surface (yellow line) at one location in medial prefrontal cortex. Note that while the brain-scalp distance is shown on a two-dimensional slice for illustration, all distances were calculated using the minimum distance in three dimensions. C. Enlargement of posterior portion of mid-sagittal slice of newborn brain. D. Lateral view of newborn brain hull surface model. Colors indicate distance between brain and scalp at each brain location (color scale shown below). Lack of color indicates regions with ill-defined brain-scalp distance in inferior regions of the brain. E. Superior view of newborn brain hull surface model. F. Mid-sagittal slice through a child brain (subject CBB, age 7 years). G. Enlargement of anterior portion of mid-sagittal slice of child brain. Same scale as (B). H. Enlargement of posterior portion of mid-sagittal slice of child brain. I. Lateral view of child brain hull surface model. The brain is shown to the same scale as the newborn brain in (D). J. Superior view of brain hull surface model. Red colors indicate greatest distance between brain and scalp.

It is important to note that all distance calculations were performed in three dimensions. While MRI data is commonly visualized as two-dimensional slices (*e.g.* Figure 1A), electromagnetic radiation, such as the infrared light used for fNIRS, propagates in three dimensions. Calculation of distance from slice views would lead to overestimation of the brain-scalp distance, because a closer distance could be found out-of-plane, unless the slice plane is precisely aligned with the geodesic. To allow accurate 3-D distance calculations, measuring the minimum brain-scalp distance that would actually be travelled by infrared light, a surface model of the scalp was created for each subject from the T1-weighted MRI using *3dSkullStrip* and visualized using SUMA. The volumes were manually edited as needed using the *Draw Dataset* plug-in of AFNI and the *IsoSurface* program to extract the scalp boundary (yellow contour in Figure 1A).

### Brain-Scalp Distance at all Brain Locations

For two subjects (shown in Figure 1) the brain-scalp distance was calculated at all brain locations. A brain hull surface was created to represent the envelope model of the brain using *3dSkullStrip* and *IsoSurface* (green contour in Figure 1A). This 2-manifold convex hull surface is akin to a shrink wrap of the brain: the wrapping surface closely hugs the brain where it is convex and is taut where the brain is concave. For each node on the brain hull, we searched for the intersection along the direction of the surface normal at that node and the scalp surface using *SurfToSurf*. The distance from the node on the convex hull to the intersection point on the scalp surface is the brain to scalp distance. The brain-scalp distance is poorly defined in places where the cortex is very far from the scalp, such as on the ventral surface of the brain. The distance is also meaningless in locations with scalp discontinuities, such as the ear pinna and ear canal, so brain-scalp distances in these locations were not analyzed (Figure 1).

### Selection of Manual Landmarks

**Figure 2:**
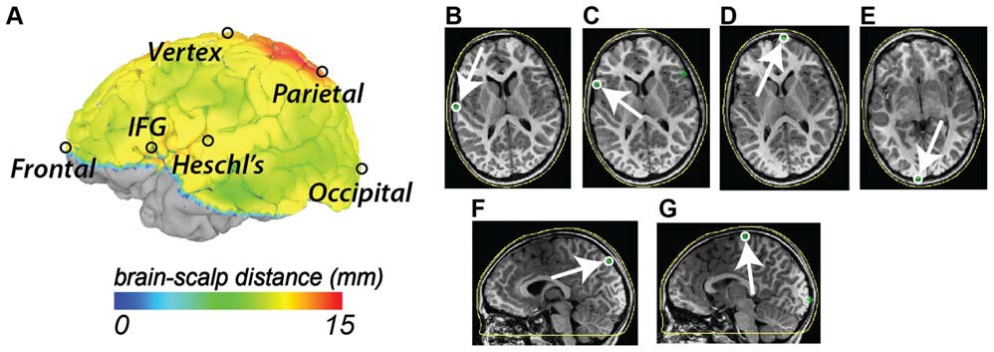
Selection of anatomical landmarks. A. Lateral view of pial surface of left hemisphere. Color scale indicates distance between brain and scalp. Black circles indicate location of anatomical landmarks (IFG, inferior frontal gyrus *par triangularis*). B. Location of Heschl's gyrus anatomical landmark shown on axial slice. Green symbol inside white circle indicates landmark. White arrow highlights location. Yellow curve shows reconstruction of scalp surface. C. Location of inferior frontal gyrus *par triangularis* landmark on axial slice. D. Location of frontal pole landmark on axial slice. E. Location of occipital pole landmark on axial slice. F. Location of parietal landmark (superior and posterior most portion of parieto-occipital sulcus) on sagittal slice. G. Location of vertex landmark on sagittal slice.

As calculation of brain-scalp distance at all brain locations was labor intensive, for the remaining subjects we calculated brain-scalp distance for a limited number of manually-selected brain locations. In order to analyze brain landmarks of interest to developmental neuroimagers, especially those using fNIRS, we searched the published experimental fNIRS literature in March 2011, using keyword searches of 11 databases. Based on the results of the literature search, we selected nine cortical landmarks that spanned most of cortex and could be reliably identified on the T1-weighted MRI scan (Figure 2). The first landmark was the occipital pole. The occipital pole was selected as the most posterior location in occipital lobe, which is of interest for fNIRS studies of visual function [20], [21], [22], [23], [24], [25], [26], [27], [28], [29], [30], [31], [32], [33], [34], [35], [36], [37]. The left and right parieto-occipital sulcus (POS) landmarks were defined as the posterior and superior edge of the left and right POS, the most posterior location on the medial surface of the precuneus of the parietal lobe [38]. The vertex landmark was defined as the most superior location in the cerebral cortex, and commonly falls in the precentral gyrus. This is the location of primary motor cortex, and is important for neuroimaging studies of motor function [39], [40], [41], [42]. The left and right Heschl's gyrus landmarks were chosen as the lateral-posterior edge of left and right Heschl's gyrus (HG), where it intersects the posterior lateral surface of the temporal lobe. HG contains core and belt areas of auditory cortex, and is especially important for developmental neuroimaging studies of auditory function and language [25], [28], [38], [43], [44], [45], [46], [47], [48], [49], [50], [51], [52], [53], [54], [55], [56], [57], [58], [59], [60]. The left and right inferior frontal gyrus (IFG) landmarks consisted of the most lateral portion of the *pars triangularis*. The left IFG landmark is near the cortical location of Broca's area, a critical node in the language network. The frontal pole landmark consisted of the most anterior location on the cerebral cortex, and was typically located near the intersection of superior frontal gyrus and the orbital gyri, relevant for frontal and prefrontal fNIRS studies of cognition [22], [23], [26], [29], [32], [40], [56], [57], [58], [61], [62], [63], [64], [65], [66], [67], [68], [69], [70], [71], [72], [73], [74], [75], [76], [77], [78], [79], [80].

### Landmark Analysis

Scalp surface models were created as detailed above. For each manually-selected landmark in each subject, the *SurfaceMetrics* program was used to calculate the Euclidean distance between the landmark and the nearest location on the scalp surface in three dimensions. The location of the closest point on the scalp surface was manually verified for each landmark. For some landmarks in some subjects, the scalp surface could not be reconstructed accurately, a failure that was easily detectable during the manual verification step. As landmark-to-scalp distance could not be calculated without an accurate scalp reconstruction, these landmarks were excluded from the remainder of the analysis. In particular, the raw brain volumes from the NIH dataset were treated with a de-identifying procedure that removed potentially identifying facial features [81]. The de-identified datasets were missing a substantial amount of scalp and adjacent soft tissue from the MRI image near the front of the head, preventing calculation of the brain-scalp distance for the frontal pole landmark for these datasets.

For analysis of variance (ANOVA), the *Matlab* routine *anovan* was used. The dependent variable was the distance between the landmark and the scalp surface and the factors were age, handedness and landmark location. To analyze age effects, subjects were grouped into 3 age groups: 0–18 months inclusive; 19 months–5 years inclusive; 6 years–12 years inclusive. The subjects in dataset 2 were right-handed according to the Edinburgh handedness inventory [82]. For the subjects in dataset 3, handedness was assessed as “the hand most commonly used to hold a pencil.” The ANOVA with handedness as a factor was performed only on children for whom handedness data was available. Degrees of freedom are reported for each statistical test, along with statistical significance.

**Figure 3:**
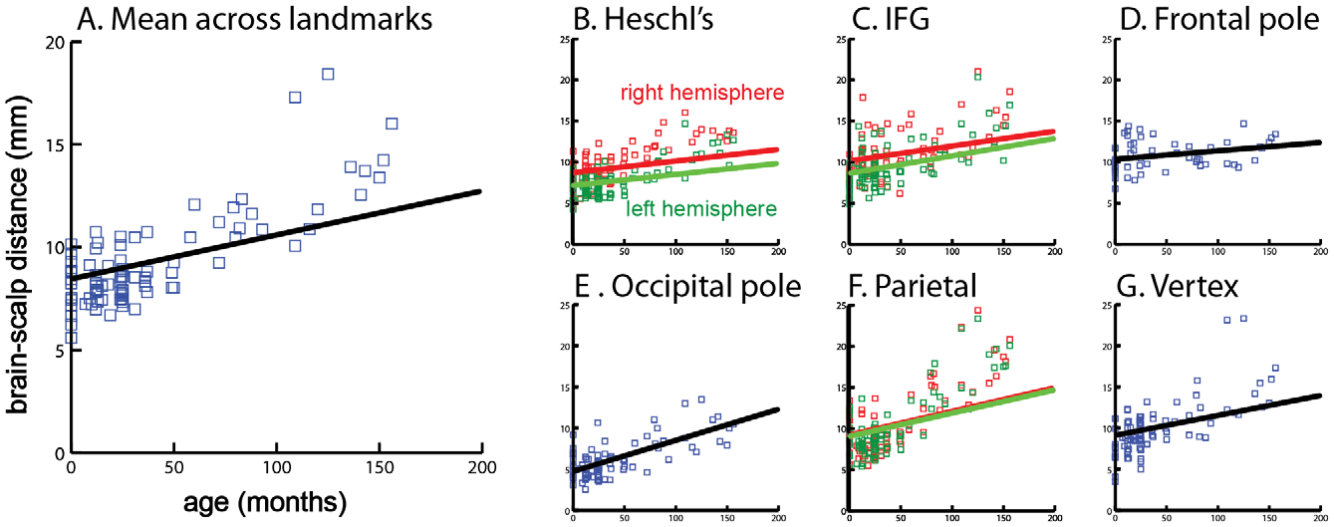
Growth charts for brain-scalp distance. A. Mean growth chart averaged across all brain landmarks. Black line indicates best-fit growth function *D* = a * t+b where *D* is brain-scalp distance in mm (y – axis) and t is age in months (x – axis). Each blue square shows average brain-scalp distance in one subject. Fit parameters are a = 0.021, b = 8.5, *r^2^* = 0.15, F_1,88_ = 16, p = 0.0001. B. Growth chart for Heschl's gyrus. Axis labels are the same as (A). Green squares indicate brain-scalp distance for left Heschl's gyrus in each individual subject and best-fit line; red squares indicate right Heschl's gyrus and best-fit line. Left hemisphere fit parameters are a = 0.013, b = 7.2, *r^2^* = 0.08, F_1,82_ = 7, p = 0.01. Right hemisphere fit parameters are a = 0.014, b = 8.7, *r^2^* = 0.06, F_1,84_ = 5, p = 0.02. C. Brain-scalp distance across age for left and right inferior frontal gyrus *par triangularis*. Left hemisphere fit parameters are a = 0.021, b = 8.75, *r^2^* = 0.1, F_1,87_ = 10, p = 0.002. Right hemisphere fit parameters are a = 0.018, b = 10.2, *r^2^* = 0.07, F_1,77_ = 6, p = 0.02. D. Growth chart for frontal pole landmark. Fit parameters are a = 0.010, b = 10.4, *r^2^* = 0.08, F_1,52_ = 4, p = 0.04. E. Growth chart for occipital pole landmark. Fit parameters are a = 0.038, b = 4.7, *r^2^* = 0.48, F_1,86_ = 80, p = 10^−13^. F. Growth chart for left and right parieto-occipital sulcus landmarks. Left hemisphere fit parameters are a = 0.028, b = 9.1, *r^2^* = 0.09, F_1,86_ = 9, p = 0.004. Right hemisphere fit parameters are a = 0.029, b = 9.3, *r^2^* = 0.09, F_1,88_ = 9, p = 0.004. G. Growth chart for vertex landmark. Fit parameters are a = 0.024, b = 9.1, *r^2^* = 0.10, F_1,85_ = 9, p = 0.003.

To provide a description of the relationship between age and scalp-brain distance, a linear function was fit to the data *D* = a * t+b where *D* is the scalp-brain distance, *t* is the age in months, and *a* and *b* are free parameters (Figure 3).

### Intervals within the brain-scalp distance

**Figure 4:**
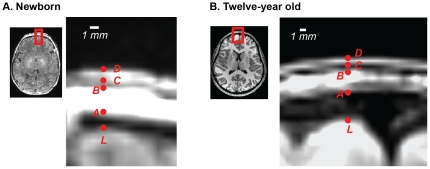
Components of brain-scalp distance. **A. Enlarged axial slice through a newborn infant brain (subject FT2009, age 2 days).** White shows scale bar. Red letters show manually selected landmarks. *L*: Frontal pole landmark (outer boundary of cerebral cortex). *A*: Inner boundary of cranial bone marrow. *B*: Outer boundary of cranial bone marrow. *C*: Inner boundary of cutis. *D*: Outer boundary of cutis. **B. Enlarged axial slice through a child brain (subject CBP, age 12 years).** White shows scale bar. Red letters show manually selected landmarks.

The scalp-brain distance contains multiple intervals, including the CSF, the meninges, the cranium (including the compact bone and the cranial bone marrow) and the skin and subcutaneous fat of the scalp (cutis). As shown in Figure 4, only some of these intervals can be distinguished on T1-weighted MRI. The cranial bone marrow in the diploic space is hyperintense on T1-weighted MRI due to its high fat content and is visible in both infants and children. The cutis is also visible as a single hyperintense band. The other compartments are not distinguishable on T1-weighted MRI. We selected four points within the scalp-brain distance adjacent to each manually-selected landmark, *L* (Figure 4). The first point (*A*), was the inner margin of the cranial bone marrow. The second point (*B*) was the outer margin of the cranial bone marrow. The third point (*C*) was the inner margin of the cutis. The fourth point (*D*) was the outer margin of the cutis. The interval *L-A* contains the meninges, the CSF and the inner table of the cranium. The two contiguous compartments of CSF and the inner table cannot be differentiated on T1-weighted MRI as both are dark. The interval A–B represents the thickness of the cranial bone marrow in the trabecular bone. The interval B–C represents the thickness of the outer table of the cranium. The interval C–D represents the thickness of the cutis.

Manual selection of these four points was performed for 20 subjects, 10 subjects from *Dataset 1* (newborns) and 10 subjects from *Dataset 2* (children). All datasets were interpolated to 0.1 mm resolution before selection of these intervals to permit precise marking. All points for a given landmark were selected in a single plane: axial for the occipital and frontal poles; coronal for the vertex; and sagittal for the parietal landmarks. The IFG and HG landmarks were not processed because the temporalis muscle interfered with identification of the cranial bone marrow and cutis. As we were unable to create a surface model of the inner and outer boundaries of the cranial bone marrow, interval measurements were done only in two-dimensions, not the more accurate three-dimensions used for total brain-scalp distance. This means that the interval measurements are an over-estimate of the true distance, but provide a rough estimate of developmental changes in the composition of the brain-scalp distance.

## Results

### Whole-brain results

For two subjects (one full-term newborn and one seven years old) brain-scalp distance was calculated at all brain locations. There were large differences in brain-scalp distance between the two subjects (Figure 1), with the older child showing larger brain-scalp distances throughout the brain. The mean brain-scalp distance across the entire brain surface was 5.9±1.5 mm (mean ± SD) for the newborn and 10.1±1.9 mm for the 7-year old. There were also large differences in brain-scalp distance between different brain regions, with areas near the vertex displaying the greatest distance. In the newborn, the mean distance was 7.6±0.4 mm for regions near the vertex compared with 4.9±0.5 mm for regions near the occipital pole. In the 7-year old, the distance was 12.9±0.7 mm for the vertex compared with 9.4±1.0 mm for the occipital pole.

### Anatomical Landmark Analysis

For the remaining subjects, brain-scalp distance was calculated for nine representative brain landmarks (Figure 2). An ANOVA was performed with three factors, age (grouped into 3 age ranges: 0–18 months, 19 months–5 years inclusive; 6 years–12 years inclusive), landmark, and handedness. Significant effects were found for age (F_2,707_ = 234, p = 10^−78^) and landmark (F_8,707_ = 44, p = 10^−57^), with significant interactions between age and landmark (F_16,707_ = 8, p = 10^−17^).

As shown by the summary data (Table 1), the main effect of age was driven by larger scalp-brain distances in older subjects for most landmarks. The main effect of landmark was driven by significantly greater distances in parietal and occipital regions. To better understand how brain-scalp distance changed with age, we constructed growth charts similar to those used for other anthropometric variables for the mean scalp-brain distance across landmarks for each subject and for each individual landmark (Figure 3). Growth curves were fit separately for each landmark, allowing the age and brain area of interest to be used to calculate a rough estimate of brain-scalp distance in future studies of subjects for whom an MRI is not available (all fit parameters provided in Figure 3 legend). The interaction between age and landmark is visible in the growth curves. Each landmark showed significant increases with age, with the steepest growth curve observed in parietal and occipital lobes (*r^2^* = 0.48, F_1,86_ = 80, p = 10^−13^). The frontal pole brain-scalp distance showed the least change with age.

**Table 1:** Mean values at each landmark.

	Occipital		L Parietal		R Parietal		Vertex		L Heschl's		R Heschl's		L IFG		R IFG		Frontal	
Age Group	μ	±SD	μ	±SD	μ	±SD	μ	±SD	μ	±SD	μ	±SD	μ	±SD	μ	±SD	μ	±SD
Infants (0–18 mos)	**5.19**	*1.45*	**8.38**	*2.01*	**8.95**	*2.24*	**8.38**	*2.10*	**6.78**	*1.16*	**8.00**	*1.67*	**8.42**	*2.09*	**9.82**	*2.60*	**10.52**	*2.01*
Younger (19 mos–5 y)	**5.60**	*1.48*	**8.62**	*1.53*	**8.62**	*1.80*	**9.48**	*1.85*	**7.08**	*1.22*	**8.82**	*1.56*	**9.00**	*1.93*	**10.35**	*2.39*	**11.04**	*2.02*
Older (6–12 y)	**9.22**	*2.25*	**16.29**	*3.67*	**16.29**	*3.71*	**13.86**	*4.10*	**10.54**	*2.02*	**12.86**	*1.55*	**12.44**	*3.11*	**13.31**	*3.10*	**11.20**	*1.51*

Numbers represent the mean (μ) brain-scalp distance at each landmark in mm (± SD).

At all ages, there were significant laterality effects in some landmarks. The greatest laterality effect was observed in Heschl's gyrus, with a significantly greater distance in right compared with left Heschl's gyrus beginning at birth and continuing through development (averaged across ages, 9.3 mm vs. 7.7 mm, p = 10^−5^). A greater brain-scalp distance was also observed in right compared with left inferior frontal gyrus (10.9 mm vs. 9.5 mm, p = 0.002) but not between right and left parietal landmarks.

### Components of brain-scalp distance

We estimated the thickness of four intervals within the brain-scalp distance: the CSF and inner table (*L to A* in Figure 4), the cranial bone marrow, the outer table of the cranium, and the cutis (subcutaneous fat and skin) for newborns and children aged 5–12 years from *Datasets 1* and *2* (Figure 4). An ANOVA was performed with two factors, age (grouped into 2 age ranges: newborns and children aged 5 years–12 years inclusive) and interval. Significant effects were found for age (F_1,392_ = 76, p = 10^−16^) and interval (F_3,392_ = 158, p = 10^−66^), with a significant interaction (F_3,392_ = 25, p = 10^−14^).

All four intervals showed a significant increase with age. The largest increase was in the CSF and inner table, with an increase from 3.4±1.9 mm (mean ± SD) in newborns to 7.0±2.7 mm in children (unpaired t-test, t_98_ = 7.4, p = 10^−10^). Smaller increases were observed in the cutis (1.2±0.5 mm *vs.* 1.5±0.49 mm, t_98_ = 3.6, p = 0.0005), the outer table (0.8±0.7 mm *vs.* 1.5±1.2 mm, t_98_ = 3.3, p = 0.001) and the cranial bone marrow (2.2±1.1 mm *vs.* 2.8±1.7 mm, t_98_ = 2.2, p = 0.03).

## Discussion

Our examination of brain-scalp distance was motivated by the desire to use fNIRS to examine auditory and language function in patients that spanned a wide age range [7]. As fNIRS depends on the transmission of infrared photons through the skull and brain tissues, changes in brain-scalp distance present an additional confounding variable that is little understood. We found significant differences between brain regions, with the greatest brain-scalp distance over parietal regions, and the smallest difference over more inferior regions of the occipital and temporal lobes. There was a significant effect of laterality in some brain areas, with greater brain-scalp distance in right compared with left temporal and frontal regions. Across landmarks, brain-scalp distance increased with age, with the exception of the frontal pole, where it stayed relatively constant.

Our findings reflect differences in brain-scalp distances between parietal and temporal regions that are consistent with previous studies of adults [8], [9], [10] and children [11], [12]. In particular, the hemispheric asymmetry that we observed in temporal regions is interesting given the importance of this region for language. In newborns, the left hemisphere is larger than the right hemisphere, especially in the temporal lobes, likely related to left-hemisphere dominance in speech and language [83], [84], [85]. Therefore, one possible explanation for the observed asymmetry in brain-scalp distance is that the skull is symmetric, but that left hemisphere brain growth results in a reduced distance between brain and the inner margin of the skull. Once established, asymmetries could remain due to the push-pull relationship between brain growth and skull expansion. For instance, hydrocephalus leads to increased intracranial pressure, causing an abnormally large skull (macrocephaly) [86]. While head circumference and brain volume are related [87] endocranium shape changes are not driven exclusively by brain growth [88]. Therefore, scalp-brain distance changes with age are likely due to a complex interplay of neural development and bone growth [89], [90], [91], [92].

### Implications for fNIRS Studies

A recent comparison of fMRI and fNIRS based on simultaneous measurements in both imaging modalities [8] found that the correlation between fMRI and fNIRS is negatively correlated with the scalp brain distance. At increasing scalp brain distances, there was much larger variance in the fNIRS signal and correspondingly weaker correlation of fNIRS with fMRI (used as a reference because it does not depend on transmission through the scalp and skull). These findings have important implications for interpreting and performing fNIRS studies of children. The optimal emitter-probe distance in fNIRS is proportional to the depth of the tissue from which the experimenter wishes to record. Therefore, our findings suggest that wider emitter-detector distances should be used in studies of parietal lobe and sensorimotor cortex because the brain-scalp distance is greater there, since the signal-to-noise of the fNIRS signal depends on brain-scalp distance [57], [93], [94], [95], [96]. Many infant fNIRS studies draw conclusions based on the change in concentration volume compared with baseline. For instance, more significant fNIRS responses in the left compared with right hemisphere are interpreted as a left-lateralized response to language [44], [52]. However, our results show that the brain-scalp distance is significantly shorter in the left hemisphere than in the right hemisphere, a potential confounding factor. Even weak responses in right hemisphere may represent a robust response [46], [47], [50]. Our results also suggest caution in interpreting changes with age: decreases in responses to a stimulus in older children could indicate brain plasticity or simply an increase in brain-scalp distance. There was a high degree of variability in brain-scalp distance across landmarks within individual subjects, and for the same landmark across subjects. This variability will be additive with inter-subject differences in brain function, making group comparisons more difficult.

Most of the increase in brain-scalp distance with age was attributable to increases in the CSF and inner table distance. This is relevant because CSF has a scattering coefficient that is an order of magnitude less than other tissues within the brain-scalp interval. Therefore, CSF can act as a waveguide for the infrared light used in fNIRS, blurring the signal and reducing spatial resolution [97]. To better understand these effects, finite or boundary element models can be constructed [98], [99]. Using these models together with the known fNIRS detector-sensory geometry, it is possible to more accurately infer hemoglobin concentration changes and hence brain activity differences [11], [12], [97].

Changes in distance between brain and scalp are also important for other human brain mapping techniques. In transcranial magnetic stimulation (TMS), electromagnetic radiation travels through the skull from a coil held outside the head in order to excite cortical neurons. This technique has received FDA approval for treatment of depression [100] and may be important for pediatric populations [101]. However, the electrical field induced by TMS falls off as the square of the distance from the coil. Therefore, in order to judge the optimal TMS current, it is important to have information about the brain-scalp distance: if the distance is small, low current will be sufficient to induce activity, while at greater distances, the same TMS current may not evoke any activity. One study estimated that for each additional mm between the scalp and brain, an additional 3% of TMS stimulator output is required to induce an equivalent motor response [9].

In the present manuscript, we selected brain landmarks of interest (such as Heschl's gyrus) and calculated the distance to the nearest point on the scalp. It is also possible to reverse this process and pick a point on the scalp, such as the inion, and calculate the distance to the nearest location on the brain, or to a particular landmark (such as the posterior margin of the calcarine fissure). This approach could be useful to understand how developmental changes in the scalp and brain influence the 10–20 system used for electrode placement used in EEG or optode placement in NIRS [10], [102], [103], [104].

### Conclusions

Our study serves as a reminder of the importance to neuroscientists of non-brain head tissue. While usually ignored, these tissues can have a considerable influence on human brain mapping studies. In PET studies of anxiety, increases in temporalis muscle blood flow due to teeth clenching were wrongly attributed to increased neural activity in the temporal lobe [105], [106], [107]. In a more recent example, gamma-band electroencephalography (EEG) changes were thought to reveal information about oscillatory neuronal activity related to higher cognitive function [108], [109], [110]. However, it is now believed that the gamma-band signal originates not from the brain but from eye-muscle potentials [111], [112]. Neuroimaging of extra-brain tissue compartments can also produce useful information. For instance, the MRI signal from the orbit can be used to assess various eye movement parameters, including the saccade frequency [113] and fixation location [114].

## References

[1] Gratton G, Fabiani M (2010). Fast optical imaging of human brain function.. Frontiers in human neuroscience.

[2] Boas DA, Gaudette T, Strangman G, Cheng X, Marota JJ (2001). The accuracy of near infrared spectroscopy and imaging during focal changes in cerebral hemodynamics.. Neuro Image.

[3] Taga G, Asakawa K, Maki A, Konishi Y, Koizumi H (2003). Brain imaging in awake infants by near-infrared optical topography.. Proceedings of the National Academy of Sciences of the United States of America.

[4] Joseph DK, Huppert TJ, Franceschini MA, Boas DA (2006). Diffuse optical tomography system to image brain activation with improved spatial resolution and validation with functional magnetic resonance imaging.. Applied Optics.

[5] Huppert TJ, Diamond SG, Boas DA (2008). Direct estimation of evoked hemoglobin changes by multimodality fusion imaging.. Journal of biomedical optics.

[6] Okada E, Firbank M, Schweiger M, Arridge SR, Cope M (1997). Theoretical and experimental investigation of near-infrared light propagation in a model of the adult head.. Applied Optics.

[7] Sevy A, Bortfeld H, Huppert T, Beauchamp MS, Tonini R (2010). Neuroimaging with near-infrared spectroscopy demonstrates speech-evoked activity in the auditory cortex of deaf children following cochlear implantation.. Hearing Research.

[8] Cui X, Bray S, Bryant DM, Glover GH, Reiss AL (2011). A quantitative comparison of NIRS and fMRI across multiple cognitive tasks.. Neuroimage.

[9] Stokes MG, Chambers CD, Gould IC, Henderson TR, Janko NE (2005). Simple metric for scaling motor threshold based on scalp-cortex distance: application to studies using transcranial magnetic stimulation.. Journal of Neurophysiology.

[10] Okamoto M, Dan H, Sakamoto K, Takeo K, Shimizu K (2004). Three-dimensional probabilistic anatomical cranio-cerebral correlation via the international 10–20 system oriented for transcranial functional brain mapping.. Neuro Image.

[11] Dehaes M, Grant PE, Sliva DD, Roche-Labarbe N, Pienaar R (2011). Assessment of the frequency-domain multi-distance method to evaluate the brain optical properties: Monte Carlo simulations from neonate to adult.. Biomedical optics express.

[12] Heiskala J, Pollari M, Metsaranta M, Grant PE, Nissila I (2009). Probabilistic atlas can improve reconstruction from optical imaging of the neonatal brain.. Optics express.

[13] Evans AC (2006). The NIH MRI study of normal brain development.. Neuroimage.

[14] Saad ZS, Reynolds RC, Argall BD, Japee S, Cox RW (2004).

[15] Argall BD, Saad ZS, Beauchamp MS (2006). Simplified intersubject averaging on the cortical surface using SUMA.. Hum Brain Mapp.

[16] Cox RW (1996). AFNI: Software for analysis and visualization of functional magnetic resonance neuroimages.. Computers and Biomedical Research.

[17] Smith SM (2002). Fast robust automated brain extraction.. Human Brain Mapping.

[18] Iglesias J, Liu C, Thompson P, Tu Z (2011). Robust Brain Extraction Across Datasets and Comparison with Publicly Available Methods..

[19] Lewiner T, Lopes H, Vieira AW, Tavares G (2003). Efficient Implementation of Marching Cubes' Cases with Topological Guarantees.. Journal of graphics, gpu, and game tools.

[20] Hoshi Y, Kohri S, Matsumoto Y, Cho K, Matsuda T (2000). Hemodynamic responses to photic stimulation in neonates.. Pediatric Neurosurgery.

[21] Bartocci M, Winberg J, Ruggiero C, Bergqvist LL, Serra G (2000). Activation of olfactory cortex in newborn infants after odor stimulation: A functional near-infrared spectroscopy study.. Pediatric Research.

[22] Taga G, Asakawa K, Hirasawa K, Konishi Y (2003). Hemodynamic responses to visual stimulation in occipital and frontal cortex of newborn infants: a near-infrared optical topography study.. Early Human Development.

[23] Taga G, Asakawa K, Maki A, Konishi Y, Koizumi H (2003). Brain imaging in awake infants by near-infrared optical topography.. Proceedings of the National Academy of Sciences of the United States of America.

[24] Wilcox T, Bortfeld H, Woods R, Wruck E, Boas DA (2005). Using near-infrared spectroscopy to assess neural activation during object processing in infants.. Journal of Biomedical Optics.

[25] Bortfeld H, Wruck E, Boas DA (2007). Assessing infants' cortical response to speech using near-infrared spectroscopy.. Neuro Image.

[26] Carlsson J, Lagercrantz H, Olson L, Printz G, Bortocci M (2008). Activation of the right fronto-temporal cortex during maternal facialrecognition in young infants.. ACTA Paediatrica.

[27] Karen T, Morren G, Haensse D, Bauschatz AS, Bucher HU (2008). Hemodynamic response to visual stimulation in newborn infants using functional near-infrared spectroscopy.. Human Brain Mapping.

[28] Nakato E, Otsuka Y, Kanazawa S, Yamaguchi MK, Watanabe S (2009). When Do Infants Differentiate Profile Face From Frontal Face? A Near-Infrared Spectroscopy Study.. Human Brain Mapping.

[29] Watanabe H, Homae F, Taga G (2010). General to specific development of functional activation in the cerebral cortexes of 2- to 3-month-old infants.. Neuro Image.

[30] Wilcox T, Bortfeld H, Woods R, Wruck E, Armstrong J (2009). Hemodynamic changes in the infant cortex during the processing of featural and spatiotemporal information.. Neuropsychologia.

[31] Meek JH, Firbank M, Elwell CE, Atkinson J, Braddick O (1998). Regional hemodynamic response to visual stimulation in awake infants.. Pediatric Research.

[32] Csibra G, Henty J, Volein A, Elwell CE, Tucker L (2004). Near infrared spectroscopy reveals neural activation during face perception in infants and adults.. Journal of Pediatric Neurology.

[33] Kusaka T, Kawada K, Okubo K, Nagano K, Namba M (2004). Noninvasive optical imaging in the visual cortex in young infants.. Human Brain Mapping.

[34] Blasi A, Fox S, Everdell N, Volein A, Tucker L (2007). Investigation of depth dependent changes in cerebral haemodynamics during face perception in infants.. Physics in Medicine and Biology.

[35] Wilcox T, Bortfeld H, Woods R, Wruck E, Boas DA (2008). Hemodynamic response to featural changes in the occipital and inferior temporal cortex in infants: a preliminary methodological exploration.. Developmental Science.

[36] Villringer A, Planck J, Hock C, Schleinkofer L, Dirnagl U (1993). Near infrared spectroscopy (NIRS): A new tool to study hemodynamic changes during activation of brain function in human adults.. Neuroscience Letters.

[37] Plichta MM, Herrmann MJ, Baehne CG, Ehlis AC, Richter MM (2006). Event-related functional near-infrared spectroscopy (fNIRS): Are the measurements reliable?. Neuro Image.

[38] Suzuki M, Gyoba J, Sakuta Y (2005). Multichannel NIRS analysis of brain activity during semantic differential rating of drawing stimuli containing different affective polarities.. Neuroscience Letters.

[39] Everdell N, Gibson AP, Tullis DC, Vaithianathan T, Hebden JC (2005). A frequency multiplexed near-infrared topography system for imaging functional activation in the brain.. Review of Scientific Instruments.

[40] Remijn GB, Kojima H (2010). Active versus passive listening to auditory streaming stimuli: a near-infrared spectroscopy study.. Journal of Biomedical Optics.

[41] Tachtsidis I, Leung TS, Tisdall MM, Devendra P, Smith M (2008). Investigation of Frontal Cortex, Motor Cortex and Systemic Haemodynamic Changes During Anagram Solving.. Advances in Experimental Medicine and Biology.

[42] Steinbrink J, Villringer A, Kempf F, Haux D, Boden S (2006). Illuminating the BOLD signal: combined fMRI–fNIRS studies.. Magnetic Resonance Imaging.

[43] Zaramella P, Freato F, Amigoni A, Salvadori S, Marangoni P (2001). Brain auditory activation measured by near-infrared spectroscopy (NIRS) in neonates.. Pediatric Research.

[44] Peña M, Maki A, Kovacić D, Dehaene-Lambertz G, Koizumi H (2003). Sounds and silence: an optical topography study of language recognition at birth.. Proceedings of the National Academy of Sciences of the United States of America.

[45] Shimada S, Hiraki K (2006). Infant's brain responses to live and televised action.. Neuro Image.

[46] Homae F, Watanabe H, Nakano T, Asakawa K, Taga G (2006). The right hemisphere of sleeping infant perceives sentential prosody.. Neuroscience Research.

[47] Homae F, Watanabe H, Nakano T, Taga G (2007). Prosodic processing in the developing brain.. Neuroscience Research.

[48] Minagawa-Kawai Y, Mori K, Naoi N, Kojima S (2007). Neural attunement processes in infants during the acquisition of a language-specific phonemic contrast.. Journal of Neuroscience.

[49] Otsuka Y, Nakato E, Kanazawa S, Yamaguchi MK, Watanabe S (2007). Neural activation to upright and inverted faces in infants measured by near infrared spectroscopy.. Neuroimage.

[50] Taga G, Asakawa K (2007). Selectivity and localization of cortical response to auditory and visual stimulation in awake infants aged 2 to 4 months.. Neuro Image.

[51] Lloyd-Fox S, Blasi A, Volein A, Everdell N, Elwell CE (2009). Social Perception in Infancy: A near infrared spectroscopy study.. Child Development.

[52] Bortfeld H, Fava E, Boas DA (2009). Identifying cortical lateralization of speech processing in infants using near-infrared spectroscopy.. Developmental Neuropsychology.

[53] Nishida T, Kusaka T, Isobe K, Ijichi S, Okubo K (2008). Extrauterine environment affects the cortical responses to verbal stimulation in preterm infants.. Neuroscience Letters.

[54] Minagawa-Kawai Y, van der Lely H, Ramus F, Sato Y, Mazuka R (2011). Optical brain imaging reveals general auditory and language-specific processing in early infant development.. Cerebral Cortex.

[55] Kotilahti K, Nissila I, Makela R, Noponen T, Lipiainen L, Chance B, Alfano RR, Tromberg BJ, Tamura M, Sevick-Muraca EM (2005). Near-infrared spectroscopic imaging of stimulus-related hemodynamic responses on the neonatal auditory cortices..

[56] Grossmann T, Johnson MH, Lloyd-Fox S, Blasi A, Deligianni F (2008). Early cortical specialization for face-to-face communication in human infants.. Proceedings of the Royal Society B: Biological Sciences.

[57] Gervain J, Macagno F, Cogoi S, Pena M, Mehler J (2008). The neonate brain detects speech structure.. Proceedings of the National Academy of Sciences.

[58] Nakano T, Watanabe H, Homae F, Taga G (2009). Prefrontal Cortical Involvement in Young Infants' Analysis of Novelty.. Cerebral Cortex.

[59] Minagawa-Kawai Y, Mori K, Izumi F, Hayashi R, Sato Y (2002). Assessing cerebral representations of short and long vowel categories by NIRS.. Cognitive Neuroscience and Neuropsychology.

[60] Minagawa-Kawai Y, Mori K, Sato Y, Koizumi T (2004). Differential cortical responses in second language learners to different vowel contrasts.. Neuro Report.

[61] Sakatani K, Chen S, Lichty W, Zuo H, Wang Y (1999). Cerebral blood oxygenation changes induced by auditory stimulation in newborn infants measured by near infrared spectroscopy.. Early Human Development.

[62] Baird AA, Kagan J, Gaudette T, Walz KA, Hershlag N (2002). Frontal lobe activation during object permanence: Data from near-infrared spectroscopy.. Neuro Image.

[63] Saito Y, Aoyama S, Kondo T, Fukumoto R, Konishi N (2007). Frontal cerebral blood flow change associated with infant-directed speech.. Archives of Disease in Childhoold Fetal Neonatal Edition.

[64] Saito Y, Kondo T, Aoyama S, Fukumoto R, Konishi N (2007). The function of the frontal lobe in neonates for response to a prosodic voice.. Early Human Development.

[65] Saito Y, Fukuhara R, Aoyama S, Toshima T (2009). Frontal brain activation in premature infants' response to auditory stimuli in neonatal intensive care unit.. Early Human Development.

[66] Minagawa-Kawai Y, Naoi N, Kikuchi N, Yamamoto J, Nakamura K (2009). Cerebral laterality for phonemic and prosodic cue decoding in children with autism.. Neuro Report.

[67] Watanabe H, Homae F, Nakano T, Taga G (2008). Functional activation in diverse regions of the developing brain of human infants.. Neuro Image.

[68] Gallagher A, Theriault M, Maclin E, Low K, Gratton G (2007). Near-infrared spectroscopy as an alternative to the Wada test for language mapping in children, adults and special populations.. Epileptic Discordance.

[69] Matsuda G, Hiraki K (2006). Sustained decrease in oxygenated hemoglobin during video games in the dorsal prefrontal cortex: A NIRS study of children.. Neuro Image.

[70] Gallagher A, Bastien D, Pelletier I, Vannasing P, Legatt AD (2008). A noninvasive, presurgical expressive and receptive language investigation in a 9-year-old epileptic boy using near-infrared spectroscopy.. Epilepsy and Behavior.

[71] Ehlis AC, Herrmann MJ, Wagener AJF (2005). Multi-channel near-infrared spectroscopy detects specific inferior-frontal activation during incongruent Stroop trials.. Biological Psychology.

[72] Schecklmann M, Ehlis AC, Plichta MM, Fallgatter AJ (2008). Functional near-infrared spectroscopy: A long-term reliable tool for measuring brain activity during verbal fluency.. Neuro Image.

[73] Fallgatter AJ, Muller TJ, Strik WK (1998). Prefrontal Hypooxygenation during Language Processing Assessed with Near-Infrared Spectroscopy.. Neuropsychobiology.

[74] Herrmann MJ, Ehlis AC, Fallgatter AJ (2004). Bilaterally Reduced Frontal Activation During a Verbal Fluency Task in Depressed Patients as Measured by Near- Infrared Spectroscopy.. Journal of Neuropsychiatry and Clinical Neuroscience.

[75] Herrmann CS, Friederici AD, Oertel U, Maess B, Hahne A (2003). The brain generates its own sentence melody: a Gestalt phenomenon in speech perception.. Brain and Language.

[76] Herrmann MJ, Ehlis AC, Scheurpflug P, Fallgatter AJ (2005). Optical Topography with Near-Infrared Spectroscopy During a Verbal-Fluency Task.. Journal of Psychophysiology.

[77] Hofman MJ, Herrmann MJ, Dan I, Obrig H, Conrad M (2008). Differential activation of frontal and parietal regions during visual word recognition: An optical topography study.. Neuro Image.

[78] Liu KR, Borrett DS, Cheng A, Gasparro C, Kwan HC (2008). Near-infrared spectroscopy study of language activated hyper- and hypo-oxygenation in human prefrontal cortex.. International Journal of Neuroscience.

[79] Sakatani K, Xie Y, Lichty W, Li S, Zuo H (1998). Language-Activated Cerebral Blood Oxygenation and Hemodynamic Changes of the Left Prefrontal Cortex in Poststroke Aphasic Patients : A Near-Infrared Spectroscopy Study.. Stroke.

[80] Tanii H, Nishimura Y, Inoue K, Koshimizu h, Matsumoto R (2009). Asymmetry of prefrontal cortex activities and catechol-O-methyltransferase Val158Met genotype in patients with panic disorder during a verbal fluency task: Near-infrared spectroscopy study.. Neuroscience Letters.

[81] Bischoff-Grethe A, Ozyurt IB, Busa E, Quinn BT, Fennema-Notestine C (2007). A technique for the deidentification of structural brain MR images.. Hum Brain Mapp.

[82] Oldfield RC (1971). The assessment and analysis of handedness: the Edinburgh inventory.. Neuropsychologia.

[83] Gilmore JH, Lin W, Prastawa MW, Looney CB, Vetsa YS (2007). Regional gray matter growth, sexual dimorphism, and cerebral asymmetry in the neonatal brain.. The Journal of neuroscience : the official journal of the Society for Neuroscience.

[84] Chi JG, Dooling EC, Gilles FH (1977). Left-right asymmetries of the temporal speech areas of the human fetus.. Archives of Neurology.

[85] Hill J, Dierker D, Neil J, Inder T, Knutsen A (2010). A surface-based analysis of hemispheric asymmetries and folding of cerebral cortex in term-born human infants.. The Journal of neuroscience : the official journal of the Society for Neuroscience.

[86] Mathews MS, Loudon WG, Muhonen MG, Sundine MJ (2007). Vault reduction cranioplasty for extreme hydrocephalic macrocephaly.. Journal of neurosurgery.

[87] Lindley AA, Benson JE, Grimes C, Cole TM, Herman AA (1999). The relationship in neonates between clinically measured head circumference and brain volume estimated from head CT-scans.. Early human development.

[88] Neubauer S, Gunz P, Hublin JJ (2009). The pattern of endocranial ontogenetic shape changes in humans.. Journal of Anatomy.

[89] Ridgway EB, Weiner HL (2004). Skull deformities.. Pediatric clinics of North America.

[90] Keller B, Yang T, Chen Y, Munivez E, Bertin T (2011). Interaction of TGFbeta and BMP signaling pathways during chondrogenesis.. PloS one.

[91] Ruppe MD, Brosnan PG, Au KS, Tran PX, Dominguez BW (2011). Mutational analysis of PHEX, FGF23 and DMP1 in a cohort of patients with hypophosphatemic rickets.. Clinical endocrinology.

[92] Aldinger KA, Lehmann OJ, Hudgins L, Chizhikov VV, Bassuk AG (2009). FOXC1 is required for normal cerebellar development and is a major contributor to chromosome 6p25.3 Dandy-Walker malformation.. Nature genetics.

[93] Lloyd-Fox S, Blasi A, Elwell CE (2010). Illuminating the developing brain: The past, present and future of functional near infrared spectroscopy.. Neuroscience and Behavioral Reviews.

[94] Gervain J, Mehler J, Werker JF, Nelson CA, Csibra G (2011). Near-infrared spectroscopy: A report from the McDonnell infant methodology consortium.. Developmental Cognitive Neuroscience.

[95] Taga G, Homae F, Watanabe H (2007). Effects of source-detector distance of near infrared spectroscopy on the measurement of the cortical hemodynamic response in infants.. Neuro Image.

[96] Gratton G, Brumback CR, Gordon BA, Pearson MA, Low KA (2006). Effects of measurement method, wavelength, and source-detector distance on the fast optical signal.. Neuro Image.

[97] Custo A, Wells WM, Barnett AH, Hillman EM, Boas DA (2006). Effective scattering coefficient of the cerebral spinal fluid in adult head models for diffuse optical imaging.. Applied Optics.

[98] Ahlfors SP, Han J, Belliveau JW, Hamalainen MS (2010). Sensitivity of MEG and EEG to source orientation.. Brain Topogr.

[99] Hamalainen MS, Sarvas J (1989). Realistic conductivity geometry model of the human head for interpretation of neuromagnetic data.. IEEE Transactions on Biomedical Engineering.

[100] Hadley D, Anderson BS, Borckardt JJ, Arana A, Li X (2011). Safety, tolerability, and effectiveness of high doses of adjunctive daily left prefrontal repetitive transcranial magnetic stimulation for treatment-resistant depression in a clinical setting.. J ECT.

[101] Frye RE, Rotenberg A, Ousley M, Pascual-Leone A (2008). Transcranial magnetic stimulation in child neurology: current and future directions.. J Child Neurol.

[102] Lagerlund TD, Sharbrough FW, Jack CR, Erickson BJ, Strelow DC (1993). Determination of 10–20 system electrode locations using magnetic resonance image scanning with markers.. Electroencephalography and Clinical Neurophysiology.

[103] Reynolds GD, Richards JE (2009). Cortical source localization of infant cognition.. Developmental neuropsychology.

[104] Jurcak V, Okamoto M, Singh A, Dan I (2005). Virtual 10–20 measurement on MR images for inter-modal linking of transcranial and tomographic neuroimaging methods.. Neuro Image.

[105] Reiman EM, Raichle ME, Robins E, Mintun MA, Fusselman MJ (1989). Neuroanatomical correlates of a lactate-induced anxiety attack.. Arch Gen Psychiatry.

[106] Reiman EM, Fusselman MJ, Fox PT, Raichle ME (1989). Neuroanatomical correlates of anticipatory anxiety.. Science.

[107] Drevets WC, Videen TQ, MacLeod AK, Haller JW, Raichle ME (1992). PET images of blood flow changes during anxiety: correction.. Science.

[108] Tallon-Baudry C, Bertrand O, Delpuech C, Pernier J (1996). Stimulus specificity of phase-locked and non-phase-locked 40 Hz visual responses in human.. Journal of Neuroscience.

[109] Yuval-Greenberg S, Deouell LY (2007). What you see is not (always) what you hear: induced gamma band responses reflect cross-modal interactions in familiar object recognition.. Journal of Neuroscience.

[110] Gruber T, Muller MM (2005). Oscillatory brain activity dissociates between associative stimulus content in a repetition priming task in the human EEG.. Cerebral Cortex.

[111] Keren AS, Yuval-Greenberg S, Deouell LY (2010). Saccadic spike potentials in gamma-band EEG: characterization, detection and suppression.. Neuroimage.

[112] Yuval-Greenberg S, Tomer O, Keren AS, Nelken I, Deouell LY (2008). Transient induced gamma-band response in EEG as a manifestation of miniature saccades.. Neuron.

[113] Beauchamp MS (2003). Detection of eye movements from fMRI data.. Magn Reson Med.

[114] Laconte S, Peltier S, Heberlein K, Hu X (2006).

